# Abundances of crenarchaeal *amoA* genes and transcripts in the Pacific Ocean

**DOI:** 10.1111/j.1462-2920.2009.02108.x

**Published:** 2010-03

**Authors:** Matthew J Church, Brenner Wai, David M Karl, Edward F DeLong

**Affiliations:** 1 School of Ocean and Earth Science and Technology, Department of Oceanography, University of HawaiiHonolulu, HI 96822, USA; 2 Civil and Environmental Engineering, Massachusetts Institute of TechnologyCambridge, MA 02139, USA

## Abstract

Planktonic *Crenarchaea* are thought to play a key role in chemolithotrophic ammonia oxidation, a critical step of the marine nitrogen (N) cycle. In this study, we examined the spatial distributions of ammonia-oxidizing *Crenarchaea* across a large (∼5200 km) region of the central Pacific Ocean. Examination of crenarchaeal 16S rRNA, ammonia monooxygenase subunit A (*amoA*) genes, and *amoA* transcript abundances provided insight into their spatial distributions and activities. Crenarchaeal gene abundances increased three to four orders of magnitude with depth between the upper ocean waters and dimly lit waters of the mesopelagic zone. The resulting median value of the crenarchaeal *amoA*: 16S rRNA gene ratio was 1.3, suggesting the majority of *Crenarchaea* in the epi- and mesopelagic regions of the Pacific Ocean have the metabolic machinery for ammonia oxidation. Crenarchaeal *amoA* transcript abundances typically increased one to two orders of magnitude in the transitional zone separating the epipelagic waters from the mesopelagic (100–200 m), before decreasing into the interior of the mesopelagic zone. The resulting gene copy normalized transcript abundances revealed elevated *amoA* expression in the upper ocean waters (0–100 m) where crenarchaeal abundances were low, with transcripts decreasing into the mesopelagic zone as crenarchaeal gene abundances increased. These results suggest ammonia-oxidizing *Crenarchaea* are active contributors to the N cycle throughout the epi- and mesopelagic waters of the Pacific Ocean.

## Introduction

The physiological activities of diverse groups of planktonic microorganisms form major controls on the transformation and availability of nitrogen (N) containing compounds in the sea. Nitrification is the aerobic oxidation of ammonia (NH_3_) to nitrate (NO_3_^-^). The process occurs through two independent steps initiated by the oxidation of ammonia to nitrite (ammonia oxidation), followed by oxidation of nitrite to nitrate (nitrite oxidation). The two steps of the complete nitrification reaction are facilitated by different groups of microorganisms. Recent cultivation-dependent and independent approaches have revealed that members of the marine *Crenarchaea* appear to play an important role in ammonia oxidation, while bacterial genera, including *Nitrospina* may mediate nitrite oxidation (Francis *et al.*, 2005; Konneke *et al.*, 2005; Wuchter *et al.*, 2006; Mincer *et al.*, 2007).

Over the past two decades, non-thermophilic archaea have increasingly become recognized as abundant, ubiquitous, and dynamic components of the ocean plankton (Delong, 1992; Fuhrman *et al.*, 1992; Karner *et al.*, 2001). This recognition has stemmed in large part from advances in geochemical and molecular-based approaches to study these microorganisms. Carbon isotope analyses of archaeal lipids (Pearson *et al.*, 2001; Ingalls *et al.*, 2006), incubation-growth experiments (Ouverney and Fuhrman, 2000; Herndl *et al.*, 2005; Teira *et al.*, 2006a; Wuchter *et al.*, 2006; Kirchman *et al.*, 2007), and reconstructions of archaeal metagenomes (DeLong *et al.*, 2006; Hallam *et al.*, 2006a,b; Martin-Cuadrado *et al.*, 2008) have provided insight into marine crenarchaeal metabolism, indicating these microorganisms may rely on metabolic strategies that include chemolithoautotrophy and assimilation of organic matter. Isolation of the ammonia-oxidizing Crenarchaeon *Nitrosopumilus maritimus* (Konneke *et al.*, 2005) revealed that chemolithoautotrophic growth on ammonia resulted in stoichiometric production of nitrite. There is also evidence that marine *Crenarchaea* are capable of assimilating organic matter(Ouverney and Fuhrman, 2000; Herndl *et al.*, 2005; Kirchman *et al.*, 2007; Varela *et al.*, 2008). The relative contribution of different carbon sources and modes of metabolism may vary with depth; chemolithoautotrophic growth has been hypothesized to prevail in the upper ocean and mid-depth waters of the mesopelagic (Hansman *et al.*, 2009), with chemoorganoheterotrophy postulated to predominate in the cold, dark waters of the ocean's bathypelagic interior (Agogue *et al.*, 2008).

Despite the potential importance of *Crenarchaea* to the marine N cycle, there is only limited information available on the distributions and physiological activities of these microorganisms. In this study, we examined the transcriptional activities and distributions of presumptive nitrifying marine *Crenarchaea* sampled throughout the euphotic zone and into the mesopelagic regions (< 1000 m) across a ∼5200 km region of the Pacific Ocean. Quantitative PCR-derived estimates of crenarchaeal gene abundances indicate that *Crenarchaea* are ubiquitous components of the mesopelagic microbial assemblage. Moreover, reverse transcriptase quantitative PCR (RT-QPCR) amplification of *amoA* transcripts revealed active gene expression throughout both the epi- and mesopelagic waters of the Pacific Ocean. These data provide additional support that *Crenarchaea* actively contribute to ammonia oxidation in many different oceanic provinces, and further suggest that despite their lower abundance in the upper ocean, these microorganisms may be active in N cycling.

## Results

### Biogeochemical and hydrographic variability

**Fig. 1:**
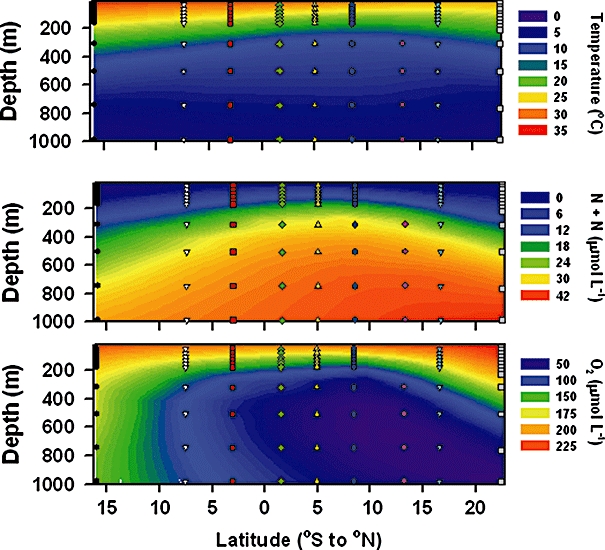
Contour plots depicting vertical and meridional distributions in ocean temperature (upper panel), nitrate + nitrite (N+N; middle panel), and dissolved O_2_ (bottom panel). Stations and depths sampled as part of this study depicted by filled symbols.

Sampling for this study included a wide range of spatially distinct oceanic environments that included well-lit, epipelagic waters to the cold, dimly lit regions of the mesopelagic zone. The most prominent meridional changes in physical and biogeochemical structure of the upper ocean occurred in the transitional region separating the oligotrophic subtropical gyres from the relatively nutrient-enriched regions of the equatorial waters. Upward doming of the thermocline in the equatorial region carried cold, nutrient-enriched, O_2_-depleted waters closer to the ocean's surface. The region of most intense upwelling (as reflected in both uplift of isotherms and nutrient distributions) occurred in the northern equatorial waters (8.6°N; Fig. 1). In contrast, upper ocean nutrient concentrations in the northern and southern subtropical gyres were very low (e.g. average N+N concentrations in the upper 100 m in these regions were generally < 100 nmol l^−1^). Among the most prominent meridional patterns observed in the waters of the mesopelagic zone (> 300 m) were changes in concentrations of N+N and dissolved O_2_, with N+N concentrations generally increasing concomitant with decreases in dissolved O_2_ concentrations from south to north (Fig. 1).

### Distributions of crenarchaeal *amoA* and 16S rRNA genes

**Fig. 2:**
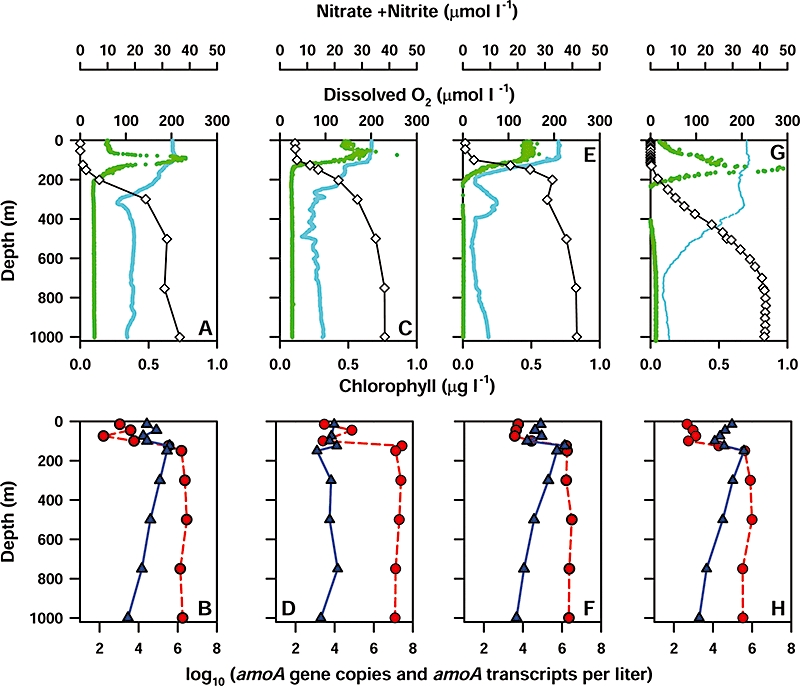
Depth-dependent variations in biogeochemical properties and crenarchaeal *amoA* gene abundance and gene transcripts at selected stations in the subtropical South Pacific (7.5°S; A, B), near-equatorial (1.7°N; C, D), northern equatorial (8.6°N; E, F), and subtropical North Pacific Ocean (16.86°N; G, H). (Top panels) Chloropigment concentrations based on *in vivo* fluorescence (green circles), N+N (open diamonds), and dissolved O_2_ (cyan circles). (Bottom panels) log_10_ crenarchaeal *amoA* genes (red circles) and *amoA* transcripts (blue triangles).

**Fig. 3:**
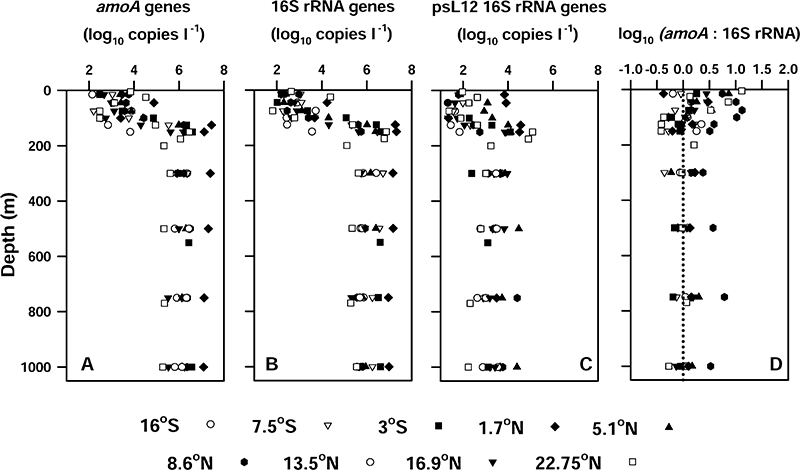
(A) Vertical profiles of crenarchaeal *amoA*, (B) MGI 16S rRNA, (C) psL12-related phylotype 16S rRNA gene abundances for all stations sampled, and (D) ratio of crenarchaeal *amoA* to total *Crenarchaea* 16S rRNA (MGI + psL12) gene copies; dotted line depicts 1:1 ratio.

Vertical profiles of QPCR-derived crenarchaeal *amoA* gene abundances varied strongly with depth. Gene abundances typically increased three to four orders of magnitude between the near-surface ocean and the lower mesopelagic zone ( Figs 2 and 3). Crenarchaeal *amoA* gene abundances in the near surface ocean ranged from 1 × 10^2^ to 7 × 10^3^ copies l^−1^, with concentrations increasing through the lower euphotic zone, and ranging from 2 × 10^5^ to 2 × 10^7^ copies l^−1^ through the mesopelagic waters (300–1000 m; Fig. 3).

Despite distinct differences in biogeochemical properties, with one exception, there were no notable differences in *amoA* gene abundances among the various stations sampled (Fig. 3). The one exception occurred within the mesopelagic waters of the station sampled closest to the equator (1.7°N). Below the euphotic zone at this station, *amoA* gene abundances were approximately an order of magnitude greater (exceeding 10^7^*amoA* copies l^−1^) than those sampled at any other station in this study (Fig. 3; Table 1).

**Table 1:** Variability in depth-averaged concentrations of nitrate + nitrite (N+N), dissolved O_2_, crenarchaeal genes (*amoA* and 16S rRNA), and crenarchaeal *amoA* gene transcripts at stations sampled for this study.

Property	16.0°S	7.5°S	3.0°S	1.7°N	5.1°N	8.6°N	13.5°N	16.9°N	22.75°N
MLD (m)	47	68	90	110	102	81	66	29	55
1% PAR (m)	ND	ND	ND	104	116	107	163	127	115
0–100 m									
N+N (μmol N l^−1^)	3 × 10^−2^	1.4 × 10^−1^	3.9	4.9	2.8	1.8	6 × 10^−3^	5 × 10^−3^	4 × 10^−3^
O_2_ (μmol O_2_ l^−1^)	209	202	203	196	202	200	213	215	213
*amoA* genes (genes l^−1^)	2.5 × 10^3^	2.3 × 10^3^	1.0 × 10^4^	2.4 × 10^4^	6.7 × 10^3^	7.5 × 10^3^	ND	8.5 × 10^2^	8.2 × 10^3^
MGI + psL12 rRNA genes (genes l^−1^)	1.8 × 10^3^	1.4 × 10^3^	1.6 × 10^4^	1.0 × 10^4^	3.9 × 10^3^	8.4 × 10^2^	ND	5.4 × 10^2^	5.2 × 10^3^
*amoA* transcripts (transcripts l^−1^)	8.1 × 10^4^	4.0 × 10^4^	1.3 × 10^5^	3.5 × 10^3^	2.5 × 10^4^	6.4 × 10^4^	ND	4.8 × 10^4^	2.5 × 10^4^
*amoA* transcripts per *amoA* gene	3.2 × 10^1^	1.8 × 10^1^	1.3 × 10^1^	1.5 × 10^−1^	3.7 × 10^0^	8.5 × 10^0^	ND	5.6 × 10^1^	3.1 × 10^0^
100–300 m									
N+N (μmol N l^−1^)	3.4	8.8	20	21	23	28	18	8.9	3.4
O_2_ (μmol O_2_ l^−1^)	214	193	170	173	151	119	173	243	207
*amoA* genes (genes l^−1^)	8.6 × 10^5^	1.6 × 10^6^	2.3 × 10^6^	1.8 × 10^7^	1.4 × 10^6^	1.6 × 10^6^	ND	4.9 × 10^5^	6.8 × 10^5^
MGI + psL12 rRNA genes (genes l^−1^)	9.9 × 10^5^	3.3 × 10^6^	2.5 × 10^6^	1.7 × 10^7^	1.7 × 10^6^	5.6 × 10^5^	ND	4.1 × 10^5^	1.9 × 10^6^
*amoA* transcripts (transcripts l^−1^)	7.1 × 10^4^	2.2 × 10^5^	3.9 × 10^5^	5.3 × 10^3^	2.1 × 10^5^	5.0 × 10^5^	ND	2.1 × 10^5^	3.1 × 10^5^
*amoA* transcripts per *amoA* gene	8.3 × 10^−2^	1.4 × 10^−1^	1.7 × 10^−1^	2.8 × 10^−4^	1.5 × 10^−1^	3.1 × 10^−1^	ND	4.3 × 10^−1^	4.5 × 10^−1^
300–1000 m									
N+N (μmol N l^−1^)	28	33	41	39	43	43	43	39	34
O_2_ (μmol O_2_ l^−1^)	182	129	77	94	66	47	29	83	119
*amoA* genes (genes l^−1^)	1.2 × 10^6^	2.5 × 10^6^	2.9 × 10^6^	2.1 × 10^7^	1.8 × 10^6^	2.9 × 10^6^	2.3 × 10^6^	7.4 × 10^5^	2.9 × 10^5^
MGI + psL12 rRNA genes (genes l^−1^)	1.2 × 10^6^	3.8 × 10^6^	3.7 × 10^6^	1.6 × 10^7^	1.9 × 10^6^	7.6 × 10^5^	6.9 × 10^5^	6.2 × 10^5^	3.0 × 10^5^
*amoA* transcripts (transcripts l^−1^)	1.8 × 10^4^	7.9 × 10^4^	1.7 × 10^5^	9.0 × 10^3^	6.1 × 10^4^	1.3 × 10^5^	2.4 × 10^4^	7.9 × 10^4^	1.5 × 10^5^
*amoA* transcripts per *amoA* gene	1.5 × 10^−2^	3.2 × 10^−2^	6.0 × 10^−2^	4.3 × 10^−4^	3.3 × 10^−2^	4.4 × 10^−2^	1.0 × 10^−2^	1.1 × 10^−1^	5.2 × 10^−1^

Depth-averaged concentrations calculated based on depth-integrated inventories divided by depth of integration for each property. No data (ND).

We also examined the vertical and meridional distributions of *Crenarchaea* based on QPCR amplification of Marine Group I (MGI) and psL12 phylotype crenarchaeal 16S rRNA genes. Similar to the distributions observed in *amoA* genes, the MGI 16S rRNA gene abundances were relatively low (10^2^−10^3^ copies l^−1^) in the upper ocean (< 100 m) and increased three to four orders of magnitude below the epipelagic layer (Fig. 3). Similar to patterns observed in *amoA* gene distributions, there were no clear regional differences in MGI crenarchaeal 16S rRNA genes, except for the large increase in gene abundances in the mesopelagic waters near the equator (1.7°N) (Fig. 3; Table 1).

Gene abundances of the psL12 crenarchaeal phylotype were consistently lower than those of the MGI, with psL12 gene abundances ranging from below detection (less than ∼20 copies l^−1^) in the upper ocean, to a maximum of 1 × 10^5^ copies l^−1^ in the mesopelagic waters (Fig. 2). Similar to the distributions of the other crenarchaeal genes, the psL12 16S rRNA abundances generally increased with depth with peak gene abundances (10^3^ to 10^4^ copies l^−1^) often occurring between 125 and 300 m depth (Fig. 3). As observed with the MGI and *amoA* gene abundances, concentrations of the psL12 phylotype were greatest at the station near the equator (1.7°N). Unlike the other phylotypes measured in this study, the psL12 gene abundances measured at this station were elevated throughout the upper ocean rather than the mesopelagic waters (Fig. 3).

Comparing the *amoA* gene abundances to the sum of the crenarchaeal 16S rRNA gene abundances (MGI + psL12) revealed that at most depths and stations, *amoA* gene abundances were very similar to 16S rRNA gene abundances (Fig. 3; Table 1). The resulting crenarchaeal *amoA* : 16S rRNA gene ratios did not vary significantly with depth (one-way analysis of variance, *P* = 0.06), although at several stations, the ratio was elevated in the upper 150 m of the ocean and declined with increasing depth (Fig. 3). The resulting *amoA* to crenarchaeal 16S rRNA gene abundance ratio ranged between 0.4 and 13 throughout the water column, with a median value of 1.3. Variability in this ratio is likely influenced by the wide range of absolute abundances of *Crenarchaea*, such that small variance where abundances were low could have large effects on the resulting *amoA* : 16S rRNA ratio.

### Crenarchaeal *amoA* transcript abundances

**Fig. 4:**
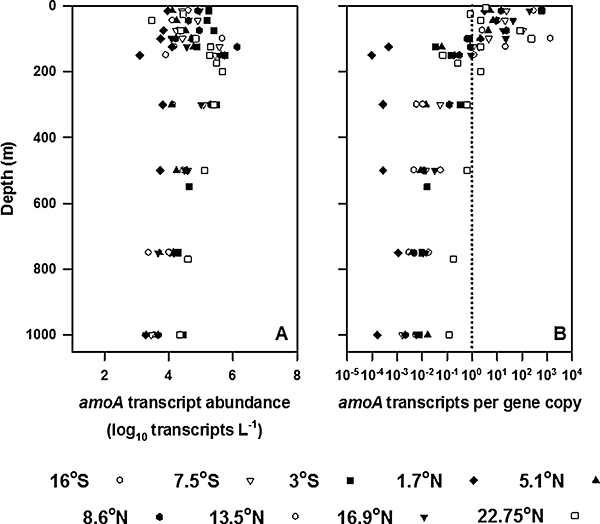
A. Vertical profiles of crenarchaeal *amoA* gene transcripts. B. Ratio of *amoA* transcripts per gene copy. Dotted line depicts 1:1 ratio.

In addition to evaluating archaeal gene copy abundances, we also examined vertical and meridional patterns of crenarchaeal *amoA* gene expression based on RT-QPCR amplification of mRNA transcripts. Relative to *amoA* gene abundances, crenarchaeal *amoA* transcripts were less variable with depth. For example, in the near surface ocean, *amoA* transcripts ranged between 9 × 10^3^ and 2 × 10^5^ transcripts l^−1^, often increasing one to two orders of magnitude in the dimly lit waters of the lower euphotic zone (100–200 m; Fig. 2). Below this zone of peak *amoA* expression, transcript abundances declined into the interior of the mesopelagic zone (Fig. 4). Among the various regions sampled, transcript abundances were greatest in the northern equatorial waters (8.6°N) where cold, N+N-enriched and O_2_ depleted waters upwelled into the upper ocean. At this station, *amoA* transcript abundances were approximately fivefold greater (> 1 × 10^6^ transcripts l^−1^) than those measured in other regions of the study site. In contrast, the lowest *amoA* transcript abundances (∼1 × 10^3^ transcripts l^−1^) were measured at the near-equatorial station (1.7°N), where *amoA* and 16S rRNA gene abundances were maximal.

One of the most notable differences between the distributions of crenarchaeal *amoA* transcripts and gene abundances occurred in the upper ocean (< 100 m) waters where the abundance of archaeal *amoA* genes was relatively low, but transcripts were relatively abundant. In this well lit region of the ocean, the gene-copy normalized *amoA* transcript abundances averaged ∼80 transcripts per gene copy (Fig. 4). The copy normalized transcript abundances approached unity in the transitional region separating the epipelagic from mesopelagic zone (125–200 m), before decreasing with depth into the lower mesopelagic zone (Fig. 4). In the mesopelagic waters *amoA* gene abundances increased coincident with decreases in gene expression resulting in low and spatially variable (ranging 1 × 10^−4^ and 6 × 10^−1^) transcripts per gene copy (Fig. 4, Table 1).

## Discussion

Quantitative PCR amplification of crenarchaeal *amoA* and 16S rRNA genes together with RT-QPCR amplification of *amoA* mRNA transcripts provided insight into the distributions and abundances of crenarchaeal *amoA* genes and transcripts across ∼5200 km of the Pacific Ocean. At all stations sampled, *amoA* gene abundances increased three to four orders of magnitude between the well-lit near-surface waters and the lower mesopelagic zone. In contrast, *amoA* transcripts tended to be elevated in the transitional waters (100–200 m) separating the epipelagic region from the mesopelagic zone. Strikingly, when normalized to gene copies, *amoA* transcription was greatest throughout the well-lit portions of the upper ocean.

To date, there is limited information on processes regulating the transcriptional activities of ammonia-oxidizing microorganisms in the sea. The observed depth-dependent patterns of gene copy normalized *amoA* expression likely reflect vertical modification of crenarchaeal growth rates deriving from changes in substrate availability, temperature, and perhaps sunlight. Laboratory studies with cultivated marine ammonia-oxidizing bacteria and natural assemblages of soil-dwelling *Crenarchaea* indicate expression of the *amo* operon is upregulated by external concentrations of ammonia (Treusch *et al.*, 2005; Berube *et al.*, 2007; El Sheikh and Klotz, 2008). Although we did not measure ammonia concentrations as part of this study, concentrations in the open ocean typically range in the 10 to 100 nmol l^−1^ (Lipschultz, 2001; Woodward and Rees, 2001; Rees *et al.*, 2006), with peak concentrations often measured in the mid to lower euphotic zone, above the depth of the primary nitrite maxima (Gruber, 2008). Our finding that *amoA* gene transcripts often peaked in the transitional region separating the euphotic zone from mesopelagic waters could be consistent with the presumed depth-dependent changes in ammonia concentrations. Temperature has also been shown to play a role in regulating crenarchaeal *amoA* transcription in terrestrial soils (Tourna *et al.*, 2008). In the present study, we observed elevated gene-copy normalized *amoA* transcript abundances throughout the warm upper ocean waters; however, we did not observe a significant relationship (least-squares linear regression, *P* > 0.05) between *amoA* transcripts per gene copy and temperature in our study.

Prior studies evaluating crenarchaeal *amoA* transcription in the sea have largely focused on regions where low concentrations of dissolved oxygen play an important role in shaping N cycling processes. For example, in the Black Sea, Lam and colleagues (2007) observed most active *amoA* transcription coincided with the vertical transition from oxic to suboxic waters. Within this depth stratum, crenarchaeal *amoA* transcripts were ∼10^4^ transcripts l^−1^ (Lam *et al.*, 2007). In the waters of the Peruvian upwelling region, crenarchaeal *amoA* transcript abundances were somewhat greater than measured in the Black Sea, ranging from 10^5^ to 10^6^ transcripts l^−1^. Peak transcript abundances in the Peruvian upwelling waters occurred coincident with the top of the nitracline, where dissolved O_2_ concentrations decreased to < 10 μmol l^−1^ and ammonium concentrations exceeded ∼1 μmol l^−1^ (Lam *et al.*, 2009). In the present study, transcript abundances ranged between 10^3^ and 10^6^ transcripts l^−1^, with peak transcript abundances occurring near the top of the nitracline in the O_2_ depleted, upwelled waters north of the equator (8.6°N). However, although dissolved O_2_ concentrations of these upwelled waters were lower than those measured at comparable depths along our transect, the O_2_ content of this water was upwards of 65 μmol l^−1^, suggesting the availability of O_2_ likely did not play a major role in structuring N cycle processes in these waters.

The observation that total *amoA* transcript abundances often peaked in the dimly lit region separating the epipelagic from the mesopelagic waters appears consistent with previously reported depth-structure in rates of marine ammonia oxidation (Wada and Hattori, 1971; Olson, 1981a; Ward, 1987; Yoshida *et al.*, 1989; Dore and Karl, 1996a). In the northern regions of the North Pacific Subtropical Gyre (NPSG), Olson (1981a) found that rates of ammonia oxidation ranged between 2.2 and 7.3 nmol N l^−1^ day^−1^, with peak rates coinciding with the vertical position of the primary NO_2_^-^ maxima of the lower euphotic zone (100–175 m). Similarly, at Station ALOHA in the central NPSG, Dore and Karl (1996a) found that rates of ammonia oxidation ranged from 1 to 134 nmol l^−1^ day^−1^ in the upper 200 m of the water, with peak rates typically occurring in the low light regions (150–175 m) of the primary nitrite maxima.

Previous studies have demonstrated inhibitory influences of sunlight on ammonia oxidation and growth of ammonia-oxidizing bacteria (Olson, 1981b; Ward, 1987; Horrigan and Springer, 1990; Guerrero and Jones, 1996). Although the exact mechanisms underlying this photoinhibition remain unknown, laboratory studies have found evidence for reversible photooxidative damage to the ammonia monooxygenase protein under short wavelength (< 410 nm) radiation (Hooper and Terry, 1974; Hyman and Arp, 1992). The influences of light on crenarchaeal ammonia oxidation activities or transcriptional responses have not yet been reported. However, the observation that the gene copy normalized *amoA* transcript abundances were elevated in the upper ocean where light intensities were upwards of 80–90% of the incident flux raises the possibility that marine *Crenarchaea* might be less sensitive to photoinhibition than their more well-studied bacterial counterparts. Alternatively, the observed depth-related changes in *amoA* transcription may not reflect changes in rates of ammonia oxidation by these microorganisms, but rather reflects post-transcriptional or translational modification of the transcribed gene product. As such, the elevated copy normalized transcriptional activities observed throughout the upper ocean could reflect increased turnover of the ammonia monooxygenase protein (perhaps compensating for photooxidative damage) rather than increases in the per cell ammonia oxidation activities. Future investigations focused specifically on examining the role of sunlight on crenarchaeal ammonia oxidation, gene transcription, as well as proteomic analyses, should provide insight to these transcriptional patterns.

Numerous studies have examined depth-dependent structure in the abundance of *Archaea* in the ocean; with several of these studies providing evidence that phylogenetically distinct populations of *Crenarchaea* vertically segregate within the epi-, meso-, and bathypelagic waters (Massana *et al.*, 2000; Francis *et al.*, 2005; Hallam *et al.*, 2006b; Mincer *et al.*, 2007; Beman *et al.*, 2008). In a recent study in the Gulf of California, Beman and colleagues (2008) measured depth distributions of crenarchaeal *amoA* genes together with ^15^N-based measurements of ammonia oxidation. These authors identified two vertically segregated groups of phylogenetically distinct crenarchaeal clades with rates of ammonia oxidation greatest in those regions where the upper ocean clade was most abundant (Beman *et al.*, 2008). Although the QPCR primers utilized in the present study did not allow discrimination of the transcriptional activities of these vertically separated *amoA*-containing crenarchaeal groups, we observed gene copy normalized *amoA* transcript abundances were greatest in the epipelagic waters, decreasing several orders of magnitude with increasing depth. This depth-dependent decrease in gene copy normalized *amoA* transcript abundances would be consistent with the presumed reduced input of ammonia accompanying vertical attenuation of particle flux into the mesopelagic zone (Karl *et al.*, 1984). However, the observation that transcriptional activity appears greatest in the upper ocean opens the possibility that despite low abundances, *Crenarchaea* might play a role in ammonia oxidation in the epipelagic layers of the ocean. Such results might shed insight into microorganisms responsible for upper ocean nitrification (Dore *et al.*, 1998; Yool *et al.*, 2007).

Over a wide range of epi- and mesopelagic habitat conditions, we generally observed close correspondence between crenarchaeal *amoA* gene abundances and total (MGI + psL12) 16S rRNA gene abundances. The median value of the *amoA* : total crenarchaeal 16S gene ratios measured in our study was 1.3, similar to previously published estimates (Wuchter *et al.*, 2006; Mincer *et al.*, 2007; Beman *et al.*, 2008). This ratio did not vary significantly with depth, although the ratio was as great as 13 at one site in the upper ocean (< 100 m), and as low as 0.4 in the lower regions of the euphotic zone (150–175 m) at another station. A recent study found that crenarchaeal *amoA* : MGI 16S rRNA gene ratios in the mesopelagic waters of the subtropical Atlantic Ocean were often < 10^−2^ (Agogue *et al.*, 2008), a finding these authors' attribute to an increasing archaeal reliance on chemoorganoheterotrophy rather than chemolithoautotrophy. In the present study, across a large region of the subtropical and tropical Pacific Ocean, we found no clear evidence of depth-dependent or regional changes in the crenarchaeal *amoA* : 16S rRNA ratios between the near-surface ocean and the mesopelagic waters of the central Pacific Ocean.

Additional constraint on *amoA* to 16S rRNA gene ratios can be obtained based on genomic and metagenomic sequences. The genome sequence of *Nitrosopumilus maritimus*, a MGI Cenarchaeum, contains a single copy of the *amoA* gene. Similarly, the *Cenarchaeum symposium* genome also contains a single gene copy of the *amoA* gene (Hallam *et al.*, 2006a). Moreover, metagenomic reconstruction of natural assemblages of *Crenarchaea* in the bathypelagic waters of the Pacific Ocean indicates that when normalized to gene length, the ratio of *amoA* to 16S rRNA genes approaches unity (Konstantinidis *et al.*, 2009).

Although the observation that crenarchaeal *amoA* : 16S rRNA gene ratios approached 1 appears consistent with genome reconstructions and metagenomic surveys, such results appear contrary to the study by Agogue and colleagues (2008) in the Atlantic Ocean. Differences in these studies may stem from the selection of QPCR primers. We examined the specificity of the QPCR primers utilized by Agogue and colleagues (2008) against a database containing > 200 publicly available crenarchaeal *amoA* gene sequences derived from both PCR clone libraries and ocean metagenomes. Consistent with the results of Konstantinidis and colleagues (2009), we found several nucleotide mismatches in these primers to the dominant *amoA* phylotypes reported from the meso- and bathypelagic waters of the Pacific Ocean.

While our results suggest the majority of *Crenarchaea* in the epi- and mesopelagic waters of the Pacific contain *amoA* genes, and thus likely possess the genetic capacity to oxidize ammonia for energy, such results do not preclude the possibility that marine *Crenarchaea* utilize diverse metabolic pathways, including chemoorganoheterotrophy, to sustain their growth. Numerous studies indicate MGI *Crenarchaea* are capable of assimilating both inorganic and organic carbon substrates during growth (Teira *et al.*, 2006b; Kirchman *et al.*, 2007; Varela *et al.*, 2008), and metagenomic analyses suggest these microorganisms have the capacity to utilize other forms of reduced N, including urea as potential sources of energy (Hallam *et al.*, 2006b). Whether or not MGI *Crenarchaea* can derive energy from any of these alternative substrates remains an open question.

In conclusion, QPCR amplification of crenarchaeal genes and RT-QPCR amplification of *amoA* gene transcripts provided information on the spatial distributions and transcriptional activities of these microorganisms in the Pacific Ocean. Crenarchaeal abundances demonstrated strong vertical structure with greatest abundances observed in the dimly lit waters of the mesopelagic zone. In contrast, crenarchaeal *amoA* gene expression was less variable with depth, and generally decreased below the euphotic zone. When normalized to gene copy abundances, the resulting patterns of transcription suggest that despite low abundances, *Crenarchaea* may be physiologically active in ammonia oxidation throughout the euphotic zone. Future studies focused on defining specific processes controlling crenarchaeal physiology (e.g. light, ammonia, temperature, dissolved oxygen) and abundance (e.g. grazing and viral infection), should provide important new insights into controls on ammonia oxidation in the sea.

## Experimental procedures

### Biogeochemical analyses

Seawater samples for subsequent biogeochemical analyses were collected from discrete depths in the upper ocean using 24 10 l polyvinyl chloride (PVC) sampling bottles attached to a Conductivity-Temperature-Depth (CTD) rosette sampling system. In total, 8–12 discrete depths were sampled from vertical profiles (0–1000 m) at nine locations between ∼16°S, 170°W and 22.75°N, 158°W in the Pacific Ocean (Fig. 1). Two stations were sampled in the warm, low chlorophyll northern waters of the South Pacific, including a station in the subtropical gyre (16°S, 170°W) and a station in the eastern portion of Western Pacific Warm Pool (7.5°S, 167°W). Samples were also collected from within the near-equatorial waters of both the North and South Pacific (3.0°S, 166°W; 1.7°N, 163°W; 5.1°N, 161°W; 8.6°N, 161°W), and at three stations in the NPSG (13.5°N, 159°W; 16.9°N, 159°W; 22.75°N, 158°W).

High resolution vertical scale (1 m) hydrographic data in the upper ocean were obtained using a Sea Bird CTD equipped with dissolved O_2_ and fluorescence sensors. The O_2_ and fluorescence sensors were calibrated against discrete measurements of dissolved O_2_ (Carritt and Carpenter, 1966) and chloropigment (chlorophyll + phaeopigment) active fluorescence (Letelier *et al.*, 1996). Seawater for determination of nutrient concentrations was subsampled into acid washed 125 ml or 500 ml polyethylene bottles and the bottles were capped and frozen upright. At the shore-based laboratory, high sensitivity measurements of NO_3_^-^+ NO_2_^-^ (N+N) were determined based on the chemiluminescence methodology developed by Garside (1982) as modified by Dore and Karl (1996b).

### Nucleic acid sampling

Seawater samples for subsequent extraction of planktonic nucleic acids were collected from 10 discrete depths spanning the epi- and mesopelagic zones (15 m to 1000 m) at eight stations located between 16°S, to 16.9°N during the CMORE-BULA (Center for Microbial Oceanography: Research and Education-Biogeochemistry Underwater: Latitudinal Assessment) research cruise (15–26 April 2007). Additional samples were collected from 12 discrete depths (15–1000 m) during a Hawaii Ocean Time-series program cruise (3–7 May 2007) to Station ALOHA (22.75°N, 158°W) in the NPSG. Seawater samples were collected using 10 l PVC bottles attached to a CTD rosette sampler. Water was subsampled from the CTD rosette into 10 l, acid-rinsed polyethylene carboys and between 2 and 4 l was immediately filtered using a peristaltic pump onto 25 mm diameter 0.2 μm pore size Supor® filters (Pall Gelman). Upon completion of filtration, the filters were removed from the filter holders and placed in 2 ml microcentrifuge tubes. Filters for subsequent RNA extraction were immersed in 500 μl RLT buffer (Qiagen RNeasy) containing 1% β-mercaptoethanol. Filters for subsequent DNA extraction were immersed in 500 μl of lysis buffer (20 mM Tris-HCL, pH 8.0; 2 mM EDTA, pH 8.0; 1.2% Triton X and 20 mg ml^−1^ lysozyme). Samples were immediately flash frozen in liquid nitrogen then stored at −80°C until processed in the shore-based laboratory.

### Nucleic acid extraction, QPCR and RT-QPCR

Planktonic RNA was extracted following the protocol described in Church and colleagues (2005). Briefly, 0.2 g of 0.1 mm glass beads was added to microcentrifuge tubes containing the sample filters and the tubes were placed inside a Fast Prep machine (Bio 101, Carlsbad, CA, USA) and agitated for 1.5 min. Following this bead beating step, tubes were centrifuged at 8500 *g* for 30 s, and the supernatants transferred to clean 2 ml microcentrifuge tubes with an equal volume of 70% ethanol. Samples were applied to Qiagen RNeasy® Mini columns (Qiagen, Valencia, CA, USA) and total RNA was purified and eluted following the manufacturer's specifications. RNA extracts were treated with DNase I following the Qiagen On-Column DNase I® RNA extraction protocol; RNA was eluted from spin column with 30 μl RNase-free water and stored frozen at −80°C. RNA concentrations were determined fluorometrically using the Quant-iT® RNA assay kit (Invitrogen, Carlsbad, CA, USA) and a Turner TD-700 fluormeter (Turner Designs, Sunnyvale, CA, USA).

Microcentrifuge tubes containing the sample filters for DNA extraction were placed in a water bath at 37°C for 1 h, after which 84 μl of proteinase K and 600 μl of lysis buffer AL (Qiagen DNeasy) were added to each sample. Samples were vortexed and placed in a hybridization oven at 70°C for 30 min. Following this incubation, 668 μl of 100% ethanol was added to each sample, and the microcentrifuge tubes were vortexed and transferred to Qiagen DNeasy spin columns. DNA was purified following the manufacturer's recommended protocols. DNA concentrations were determined using the Quant-iT® DNA assay protocol and quantified fluorometrically.

Total RNA was reverse transcribed using SuperScript® III first strand cDNA synthesis kit (Invitrogen) following the manufacturer's specifications. cDNA reactions consisted of 2–4 ng total RNA, 1 mmol l^−1^ dNTPs, 1× RT buffer, 5 mmol l^−1^ MgCl_2_, 10 mmol l^−1^ DTT, 40 U RNaseOUT (Invitrogen), 200 U SuperScript III RT, and 0.5 μmol l^−1^ of the antisense gene specific primer (*CrenAmoAModR;*Mincer *et al.*, 2007). Upon completion of the cDNA synthesis, 1 U RNase H was added to each reaction. The resulting cDNA was diluted to 50 μl total volume with nuclease-free water, and stored at −20°C until analysed by QPCR assays. An identical set of reactions minus the reverse-transcriptase (no-RT reactions) were performed for each RNA extract; these reactions served as controls to examine the potential contributions of carryover genomic DNA on the RT-QPCR amplification of the cDNA.

Crenarchaeal *amoA* transcript abundances and copy abundances of crenarchaeal *amoA* and 16S rRNA genes were examined using previously described QPCR protocols (Mincer *et al.*, 2007). The QPCR assays consisted of duplicate 25 μl reactions containing: 12.5 μl 2× SyberGreen Master Mix (Applied Biosystems, Foster City, CA, USA), 8 μl of nuclease-free water, 2 μl of environmental DNA or cDNA reaction mixes (including no-RT reactions), and 0.5 μM final concentration of both forward and reverse primers. Quantitative PCR reactions were analysed using an Applied Biosystems 7300, following the thermal cycling reaction conditions described in Mincer and colleagues (2007). Standards for the QPCR and RT-QPCR reactions for the *amoA* and MGI 16S rRNA consisted of serial 10-fold dilutions of plasmids containing amplified fragments of the targeted genes of interest. QPCR amplification efficiencies averaged 99%; 98%, 94% and 104% for the crenarcaheal *amoA* transcripts and gene copies of *amoA*, 16S, rRNA, and psL12 phylotypes respectively.
